# Inhibitory Effect and Mechanism on Antiproliferation of Isoatriplicolide Tiglate (PCAC) from *Paulownia Coreana*

**DOI:** 10.3390/molecules17055945

**Published:** 2012-05-18

**Authors:** Samil Jung, Hyung-In Moon, Jiyeon Ohk, Soonduck Lee, Chengping Li, Soo-Ki Kim, Myeong-Sok Lee

**Affiliations:** 1 Division of Biological Science and Research Center for Women’s Diseases, Sookmyung Women’s University, Seoul 140-742, Korea; 2 Department of Medicinal Biotechnology, College of Natural Resources and Life Science, Dong-A University, Busan 604-714, Korea; 3 College of Animal Bioscience and Technology, Konkuk University, Seoul 143-701, Korea

**Keywords:** antiproliferation agent, *Paulownia coreana*, isoatriplicolide tiglate

## Abstract

*Paulownia coreana* has traditionally been used as the medicine and health food in the treatment of cancer and infectious diseases. In the present study, a new antiproliferation agent, isoatriplicolide tiglate (PCAC) was isolated from the chloroform soluble fraction of the leaves of *Paulownia coreana*. The antiproliferation activities of PCAC plant extract was examined in breast and cervical cancer cell lines in a time-and dose-dependent manners. Our *in vitro* experiments showed that PCAC suppresses the cell growth and proliferation of cancer cells at a relatively low concentration (<10 µg/mL) and induces apoptosis at a high concentration (>50 µg/mL). Western blot analysis showed that concentration higher than 50 µg/mL induces a time-dependent increase in the percentage of apoptotic cells. In this case, PCAC uses both extrinsic and intrinsic pathways for the apoptosis. PCAC treatment decreased the expression of pro-caspase 8, 9, and 3, the main regulators of apoptotic cell death, in MDA-MB-231 cells, accompanied by the activation of caspase 8, 9, and 3. More importantly, PCAC inhibited the *in vitro* proliferation of six other human breast and cervical cancer cell lines. In conclusion, our data strongly suggest that PCAC acts as an antiproliferation agents particularly against breast and cervical cancers by inducing cell cycle arrest in the S/G2 phase and caspase dependent apoptosis at relatively low (<10 μg/mL) and high (>50 µg/mL) concentrations, respectively.

## 1. Introduction

**Figure 1:**
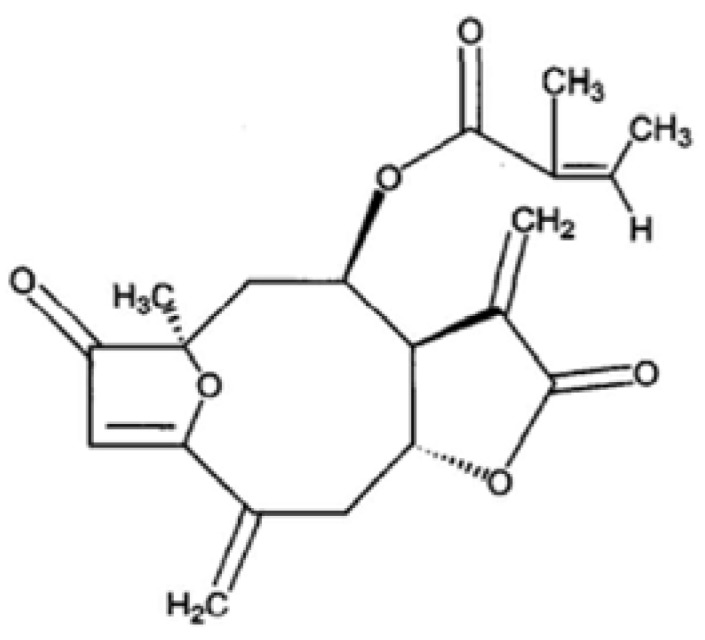
PCAC structure from *P. coreana*.

Breast and cervical cancers are the most common malignant type of cancers in women. In spite of the advanced treatments for cancerous tumors such as surgery, radiation therapy, chemotherapy, or hormone therapy, they are still an important health issue for women all over the World. After surgical removal of the tumor, radiation- and chemotherapies kill the majority of the remaining tumor cells. However, these tumors often recur due to several reasons, in which they are typically resistant to additional courses of the same therapies. Therefore, improvement in cancer treatment is required for the survival and quality of life of patients. One improvement could be achieved by the development of effective antiproliferation agents that have fewer toxic side effects than those presently available. One promising new source of therapeutic agents has been discovered in plant extracts. In fact, many cancer research studies have been conducted using traditional medicinal plants such as *P. coreana*. For example, we have previously reported that sesquiterpene lactone isolated from the flower parts of *P. coreana* has significant neuroprotective activity against glutamate-induced neurotoxicity in primary cultured rat cortical cells [1]. In this study, we report that PCAC from *P. coreana* (Figure 1) possesses antiproliferation activity against breast and cervical cancer cells.

## 2. Results and Discussion

**Figure 2:**
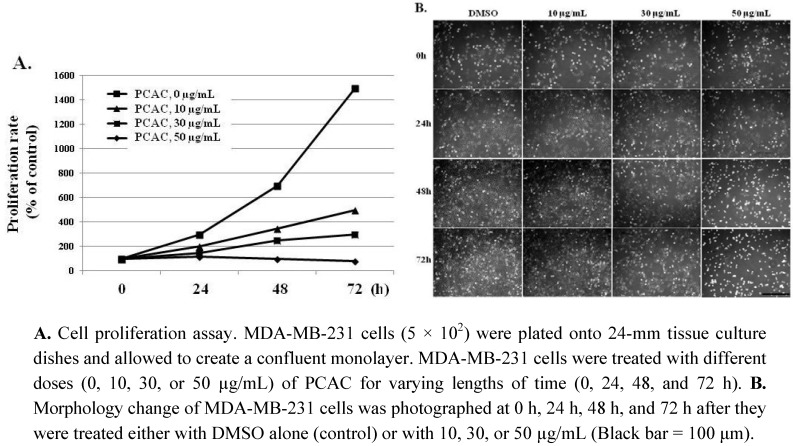
Effects of PCAC on the proliferation and viability of MDA-MB-231 cancer cells.

In an effort to find plant-derived compounds with antiproliferation activity, PCAC from *P. coreana* was tested to determine if it had antiproliferation effects on breast and cervical cancer cells. The cytotoxic activities of PCAC were at first evaluated by checking cell proliferation and viability in the MDA-MB-231 human breast cancer cell line. MDA-MB-231 cells were plated onto 24-mm tissue culture dishes and allowed to create a confluent monolayer for 24 h. These cells were then cultured in the absence and presence of various concentrations (10, 30, 50, and 100 µg/mL) of the PCAC for 0 h, 24 h, 48 h, and 72 h. PCAC showed the strongest cytotoxic effect on the cell proliferation and viability of the MDA-MB-231 cells. As shown in Figure 2, PCAC increased cell growth inhibition and cell death in a time- and dose-dependent manners compared to the control. At a relatively concentration, 10 μg/mL of PCAC, the proliferation of MDA-MB-231 cells was highly inhibited compared to the control cells. 

**Figure 3:**
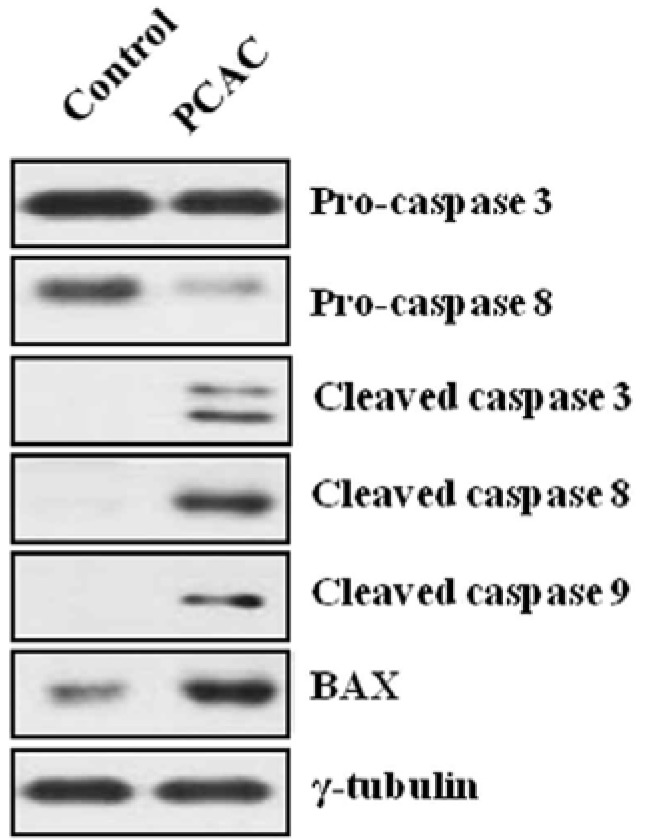
Effect of PCAC on the apoptotic cell death of MDA-MB-231 cells.

At a relatively high concentration, 50 µg/mL, the treatment with PCAC significantly increased the number of dead cells (Figure 2). PCAC treated cells exhibited typical morphologies of apoptosis, shrinkage and membrane blebbing, in which many detached and floating cells were detected in the medium. When MDA-MB-231 cells were treated with concentrations higher than 50 µg/mL of the PCAC, significant cell death of MDA-MB-231 cells was induced in 6 h (data not shown). Another representative breast cancer cell line, MCF7 cells, were also treated with different concentrations of 0, 10, 30, 50, and 100 µg/mL of PCAC and showed a similar effect as in the case of MDA-MB-231 cells (data not shown). Taken together, our results showed that PCAC from *P. coreana* inhibited cell proliferation at a low concentration and induced cell death in MDA-MB-231 breast cancer cells at a high concentration. Our previous data indicate that PCAC seems to induce apoptotic cell death at a high concentration in human breast cancer cells. Apoptosis is usually induced through the receptor-mediated extrinsic or the mitochondria-mediated intrinsic signaling pathways, which are ultimately coupled to the activation of caspases including caspase 3, 8, and 9 [2,3,4,5,6,7]. This death pathway is largely controlled by the pro-apoptotic (e.g., Bax, Bad, Bid, and Bak) and anti-apoptotic (e.g., Bcl-2 and Bcl-xL) Bcl-2 family proteins [8,9,10]. It is well known that resistance towards apoptosis is a key factor for the survival of a malignant cell. Induction of apoptosis is the most frequently observed mechanism of antiproliferation agents. Thus, one of the main goals for cancer treatment is to overcome resistance of tumor cells towards apoptosis. Therefore, we further examined the effect of PCAC on the apoptosis by analyzing the cleavage of caspase 3, 8, 9 and Bax, the crucial mediators and regulators of apoptotic pathways, in MDA-MB-231 cells using western blot analysis. As shown in Figure 3, MDA-MB 231 cells treated with 50 μg/mL of PCAC exhibited an increase in activated cleaved caspases, whereas the untreated control MDA-MB 231 cells showed no induction of caspases 3, 8 and 9 activities. PCAC treatment reduced the level of pro-caspase 3 and 8 and increased the level of cleaved caspase 3, 8 and BAX. These data suggest that PCAC induced cell death is associated with death receptor pathway. Furthermore, PCAC seems to also trigger the intrinsic signaling pathways as well as the extrinsic pathway, as indicated by up-regulation in the level of cleaved caspase 9. In a further study, it was doubted that this apoptotic cell death might to be related to the senescence. Therefore, we tested the effect of PCAC on the senescence by using a Senescence Detection Kit (BioVision, Catalog #K320–250). However, our result showed that PCAC has no effect on senescence (data not shown). Taken together, these data indicate that PCAC has an antiproliferation activity at least partly by inducing caspase-dependent apoptosis, in which PCAC seems to activate both extrinsic- and intrinsic-dependent apoptosis in MDA-MB-231 cells. We showed that PCAC suppresses cell proliferation in MDA-MB-231 cells at a relatively low concentration (10 μg/mL). To confirm the involvement of the cell cycle arrest in the inhibition of cell proliferation, a FACS analysis was exerted to check the effect of PCAC on the cell cycle in MDA-MB cells. FACS analysis shows that treatment with 10 μg/mL of PCAC induces the cell cycle arrest in the S/G2 phase in MDA-MB-231 breast cancer cell line.

**Figure 4:**
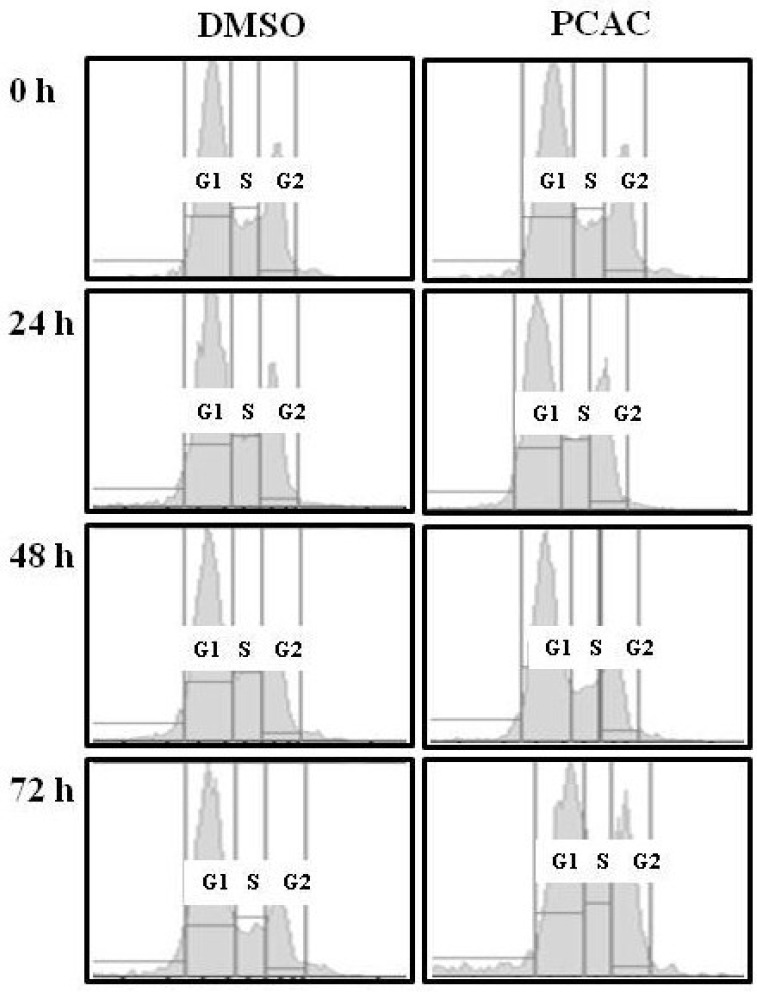
FACS analysis after the treatment of PCAC in the MDA-MB-231 cells.

**Figure 5:**
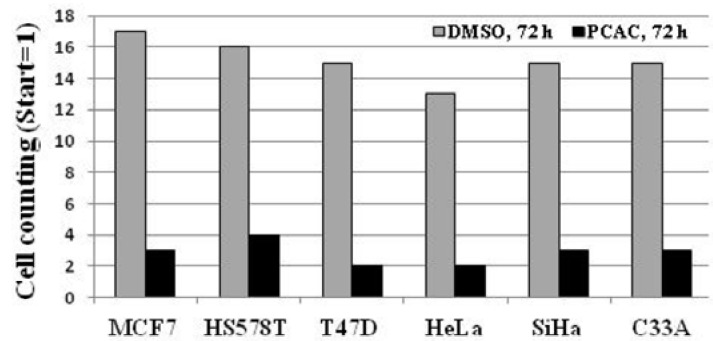
Effect of PCAC on cell proliferation of the six other cancer cell lines.

As shown in Figure 4, the untreated control cells showed normal cell cycle, whereas the treatment with 10 μg/mL of PCAC induced cell cycle arrest in the S/G2 phase after 72 h. This result suggests that treatment of low concentration of PCAC in MDA-MB-231 cells induces cell cycle arrest in the S/G2 phase, which seems to be partly responsible for the inhibition of cell proliferation. The antiproliferation activity of PCAC in MDA-MB-231 breast cancer cell line suggested the possibility of a similar activity in other cancer cell lines. Therefore, this investigation was extended to different types of breast (MCF7, HS578T and T47D) and cervical (HeLa, SiHa and C33A) cancer cell lines. We evaluated the cytotoxic effect of PCAC after Treatment with 10 µg/mL PCAC, the best concentration for the inhibition of cell proliferation in MDA-MB-231 and MCF7 cells. Cell proliferation was highly repressed in cells treated with PCAC, but not in untreated control cells (Figure 5). This result suggests that the PCAC also has high inhibitory activity on the proliferation of other cancer cells as well as on MDA-MB-231 cells. Therefore, PCAC appears to have general anti-tumoral activity in various forms of breast and cervical cancer cells. This fact implies that PCAC may be able to reduce tumor growth and proliferation when applied to treatment of various forms of other cancers. MDA-MB-231 cells were treated with either DMSO alone (control) or 50 μg/mL PCAC for 24 h. Whole cell lysates were prepared and analyzed for the activation of caspase 3, 8, 9 and Bax using Western blotting. The γ-tubulin was used as an internal control.

## 3. Experimental

PCAC was provided by Dr. Hyung-In Moon at Dong-A University (Busan, Korea). The isolation and identification of PCAC (Figure 1) was previously reported by Chung *et al*. [1]. Four breast cancer cell lines (MDA-MB-231, MCF-7, HS578T, and T47D) and three cervical cancer cell lines (HeLa, SiHa, and C33A) were grown in a DMEM medium (WelGENE Inc., Seoul, Korea). All the media were supplemented with 10% fetal bovine serum (Gibco BRL) and 1% antibiotic-antimycotic solution (Gibco BRL). All the cells were cultured at 37 °C in a humidified atmosphere composed of 95% air and 5% CO_2_. They were treated with DMSO only as a control and 10, 30, 50, or 100 μg/mL for the indicated times. For FACS analysis, the cells were harvested at the indicated time points, washed twice in an ice-cold PBS buffer, and fixed by resuspending them in absolute ethanol for 30 min. The fixed cells were centrifuged at 1,500 rpm for 5 min and washed twice with a cold PBS buffer. The cell pellets were resuspended in 0.5 mL PBS containing 50 μg/mL propidium iodide (Sigma-Aldrich Chemical Co., Seoul, Korea), 10% sodium citrate (Sigma, Seoul, Korea), 100 µg/mL RNase (Invitrogen, Seoul, Korea), and 0.001% NP40 (Sigma). Following the incubation at 37 °C for 30 min in the dark, the samples were analyzed by a FACScan flow cytometer (Becton Dickinson FACScan, Sunnyvale, CA, USA) equipped with CellQuest 3.2 software (Becton Dickinson, New Jersey, NJ, USA). The cells were centrifuged at 3,000 rpm for 3 min and washed twice in an ice-cold PBS buffer. The cells were then lased in an RIPA lysis buffer and the protein amount was quantified using a protein assay kit (Bio-Rad, Seoul, Korea). Approximately 15 μg of total protein per sample was subjected to SDS-PAGE and transferred to an Immobilon polyvinylidene fluoride (PVDF) membrane filter (Millipore, Seoul, Korea). The filter was incubated with each corresponding antibody in 5% nonfat dry milk/0.1% Tween/TBS and immunodetection was done using the ECL System (Amersham Pharmacia Biotech, Seoul, Korea). Antibodies used in this study were purchased as follows: Caspase 3 (Cell Signaling, #9662), Cleaved Caspase 3 (Asp175) (Cell Signaling, #9661), Caspase 8 and Cleaved Caspase 8 (1C12) (Cell Signaling, #9746), Cleaved Caspase 9 (Asp330) (Cell Signaling, #9501), Bax (SantaCruz, sc-20067), and γ-tubulin (SantaCruz, sc-7396). MDA-MB-231 cells were either left untreated (control) or treated with 10 μg/mL of PCAC. The various cell cycle phases are indicated. One representative experiment of three is shown. The bars represent the cell number of breast cancer cell lines (MCF7, HS578T and T47D) and cervical cancer cell lines (HeLa, SiHa and C33A) after the treatment with DMSO alone (control) or with 10 μg/mL of PCAC for 72 h.

## 4. Conclusions

In summary, our results show that PCAC isolated from the flowers of *P. coreana* possesses time- and dose-dependent antiproliferation activity in breast and cervical cancer cell lines, as indicated by its capability to induce S/G2 phase cell cycle arrest at low concentration and caspase-dependent apoptosis at a high concentration in breast and cervical cancer cell lines. PCAC appears to induce both extrinsic- and intrinsic-apoptosis. These results validate its potential use as an antiproliferation agent, particularly against breast and cervical cancer and provide important information potentially helpful in drug design and discovery. Our data imply that it can be a promising candidate for chemotherapy and chemoprevention of possibly other types of cancer cells as well as breast and cervical cancer cells. 
